# Evaluation of In Vitro Antioxidant and Anticancer Properties of the Aqueous Extract from the Stem Bark of *Stryphnodendron adstringens*

**DOI:** 10.3390/ijms19082432

**Published:** 2018-08-17

**Authors:** Débora da Silva Baldivia, Daniel Ferreira Leite, David Tsuyoshi Hiramatsu de Castro, Jaqueline Ferreira Campos, Uilson Pereira dos Santos, Edgar Julian Paredes-Gamero, Carlos Alexandre Carollo, Denise Brentan Silva, Kely de Picoli Souza, Edson Lucas dos Santos

**Affiliations:** 1 Research Group on Biotechnology and Bioprospecting Applied to Metabolism (GEBBAM), Federal University of Grande Dourados, Rodovia Dourados Itahum, Km 12, CEP: 79.804-970 Dourados, MS, Brazil; deborabaldivia@outlook.com.br (D.d.S.B.); leitefd2@gmail.com (D.F.L.); david_hiramatsu@hotmail.com (D.T.H.d.C.); jcampos_bio@yahoo.com.br (J.F.C.); uilsanto@hotmail.com (U.P.d.S.); kelypicoli@gmail.com (K.d.P.S.); 2 Federal University of Mato Grosso do Sul, University city, s/n, 79070-900 Campo Grande, MS, Brazil; paredes.gamero@gmail.com; 3 Laboratory of Natural Products and Mass Spectrometry, Federal University of Mato Grosso do Sul, University City, s/n, 79070-900 Campo Grande, MS, Brazil; carloscarollo@gmail.com (C.A.C.); denise.brentan@ufms.br (D.B.S.)

**Keywords:** Cerrado, medicinal plants, LC-DAD-MS, oxidative stress, ROS, melanoma, caspase-3, apoptosis

## Abstract

*Stryphnodendron adstringens* (Mart.) Coville (Fabaceae) is a tree species native to the Brazilian Cerrado commonly known as barbatimão. In traditional medicine, decoctions or infusions of the stem bark of this plant are used in the treatment of several diseases. The objective of this study was to analyze the chemical composition of *Stryphnodendron adstringens* aqueous extracts (SAAE) prepared from the stem bark to assess their antioxidant activity and anticancer effects as well as characterize cell death mechanisms against murine B16F10Nex-2 melanoma cells. From the SAAE, gallic acid, gallocatechin, epigallocatechin, dimeric and trimeric proanthocyanidins mainly composed of prodelphinidin units and the isomeric chromones *C*-hexosyl- and *O*-pentosyl-5,7-dihydroxychromone were identified. The SAAE showed antioxidant activity through direct free-radical scavenging as well as through oxidative hemolysis and lipid peroxidation inhibition in human erythrocytes. Furthermore, SAAE promoted apoptosis-induced cell death in melanoma cells by increasing intracellular reactive oxygen species (ROS) levels, inducing mitochondrial membrane potential dysfunction and activating caspase-3. Together, these data show the antioxidant and anticancer effects of *Stryphnodendron adstringens*. These results open new perspectives for studies against other tumor cell lines and in vivo models as well as for the identification and isolation of the chemical constituents responsible for these effects.

## 1. Introduction

Brazilian biodiversity has numerous plant species with bioactive compounds among their constituents with potential for the development of new drugs. Among them, *Stryphnodendron adstringens* (Mart.) Coville (Fabaceae) is a tree species native to the Brazilian Cerrado, commonly known as barbatimão or casca-da-mocidade. In traditional medicine, decoctions or infusions of the stem bark of this plant are used to treat prostate problems, liver and skin diseases, poor circulation, wounds, fungal infections, dental inflammation, gastritis and diabetes [1,2]. Some of these effects have already been scientifically shown, including their wound healing [3], anti-inflammatory [4,5], antifungal [6,7] activities. However, other biological properties may still be characterized.

Chemical studies show that the stem bark of *S. adstringens* has high concentrations of condensed tannins (proanthocyanidins) and flavan-3-ol monomers [8,9], which are described in the literature for their antioxidant activities and anticancer properties [10,11]. 

The search for medicinal plants with antioxidant properties has intensified in recent years [12,13,14,15,16]. Natural antioxidants are molecules that protect the organism from cellular damage resulting from excess free-radicals responsible for inducing oxidative stress [17]. 

Oxidative stress is characterized by the imbalance between the production of oxidizing substances and endogenous antioxidants, and it may cause the oxidation of biomolecules such as nucleic acids, proteins and lipids [13]. This biological condition is strongly related to the development of various diseases, including cancer [18]. 

Melanoma is the most aggressive skin cancer due to its high metastatic capacity. Melanoma cells originate in melanocytes, cells responsible for the production of melanin, which is the pigment that gives color to the skin [19]. Although melanoma is a multifactorial disease, excessive exposure to ultraviolet radiation is among the main risk factors [20]. Its worldwide incidence is increasing, with annual rates of approximately 132,000 new cases [21]. The chances of a cure are related to detection and surgical treatment in the early stages of the disease. In the more advanced stages, the five-year survival prognosis ranges from approximately 15 to 20% of cases [22]. Currently, no fully effective treatment against metastatic melanoma is available. However, different chemotherapeutic drugs are among the main melanoma treatment options [23,24]. 

Despite the benefits from the treatment of melanoma with pharmacological drugs, chemotherapeutic drug resistance and high toxicity are the main problems identified. Therefore, the identification of effective anticancer compounds and molecules with high target cell selectivity is of great pharmacological interest. Approximately 49% of Food and Drug Administration (FDA)-approved anticancer therapeutic agents are derived from natural products or their derivatives [25]. 

These chemotherapeutic agents of plant origin used in cancer treatment include vincristine, vinblastine, and Taxol [26]. Hence, the identification of compounds extracted from medicinal plants, combined with cancer treatment strategies, is crucial for developing effective therapies for melanoma. 

Thus, the objectives of this study were to analyze the chemical composition of *S. adstringens* aqueous extracts prepared from stem bark and to assess their antioxidant activity, anticancer effects and in vitro cell death mechanisms against B16F10Nex-2 melanoma cells.

## 2. Results

### 2.1. Identification of the Constituents from the SAAE by LC-DAD-MS/MS 

The chemical constituents from the *Stryphnodendron adstringens* aqueous extracts (SAAE) were identified based on UV, accurate MS and MS/MS data compared to spectral data reported in the literature, and some compounds could be confirmed by analyses of authentic standards. All identified compounds and spectral data are summarized on Table 1.

**Figure 1:**
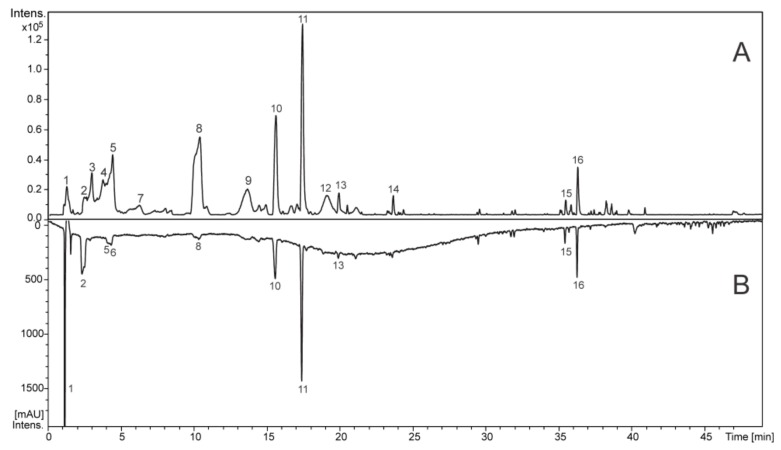
Total ion chromatogram in the negative ion mode (**A**) and chromatogram at wavelengths of 270-330 nm (**B**) of *S. adstringens* aqueous extract (SAAE).

**Figure 2:**
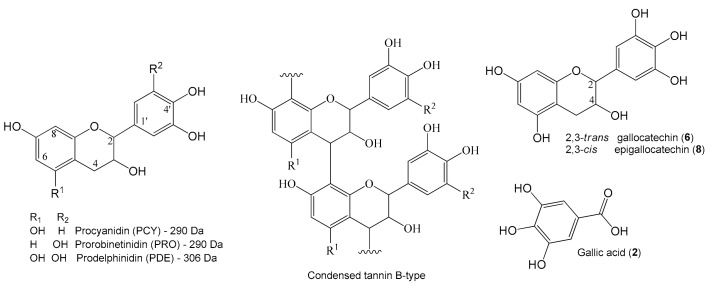
Structures of flavan-3-ol monomers composed the proanthocyanidins from *S. adstringens* aqueous extract (SAAE), a typical condensed tannin B-type and some chemical compounds that were identified.

The chromatographic peaks (Figure 1) **1**–**9** and **12**–**13** showed an intense band at ≈280 nm (λ_max_) in the UV spectrum, which are assignable to gallic acid and flavan-3-ol units, including the proanthocyanidins (condensed tannins) (Figure 2). The compounds **2**, **6** and **8** exhibited deprotonated ions at *m*/*z* 169.0140, 305.0673 and 305.0673, respectively, which are compatible with the molecular formulas C_7_H_6_O_5_ and C_15_H_14_O_7_, and these compounds were confirmed to be gallic acid, gallocatechin and epigallocatechin by the injection of standards. Their fragmentation profiles were compatible with published data [27], and they had already been reported from *S. adstringens* [8,28].

The metabolites **1**, **3**–**5**, **7**, **9** and **12** exhibited ions at *m*/*z* 609 and 593, which correspond to C_30_H_26_O_14_ and C_30_H_26_O_13_, characterizing dimeric proanthocyanins. All of the metabolites showed fragment ions at *m*/*z* 305 that are yielded from quinone methide reactions with losses of 304 and 288 *u*, thus confirming prodelphinidin (PDE) and procyanidin (PCY)/prorobinetidin (PRO). The common losses by Retro Diels Alder (RDA) reactions of 168 and 152 *u* suggest the presence of prodelphinidin/prorobinetidin and procyanidin, respectively. For example, the product ion *m/z* 423 [M – H-168-H_2_O]^−^ is observed for the metabolites **1**, **3**–**5** and **7**, the ion *m*/*z* 423 [M – H-152-H_2_O]^−^ is observed for **12** and the ion *m*/*z* 425 [M-H-168]^−^ of **9** suggested prodelphinidin (PDE), procyanidin (PCY) and prorobinetidin (PRO) units. Thus, the dimers PDE-PDE, PCY-PDE and PRO-PDE could be identified in the extract, and their spectral data are compatible with the published data [27]. In the same way, the trimer of prodelphinidin (**1**) and the hydroxy-benzoyl dimer of prodelphinidin, which already were isolated from *S. adstringens* [8], were identified.

Compounds **10** and **11** showed similar UV spectra with bands at wavelengths of approximately 257, 290 and 325 nm (shoulder), which are compatible with chromone compounds [29]. They presented accurate masses (*m*/*z* 485.1312 and 485.1317 [M − H]^−^) that corresponded to the molecular formula C_21_H_26_O_13_. The fragmentation of ions at *m*/*z* 485 yielded ions at *m*/*z* 353 [M−H-132]^−^, suggesting an *O*-pentosyl substituent on the structure. In addition, the product ions *m*/*z* 263 [M−H-90]^−^, 245 [M−H-90−H_2_O]^−^, 233 [M−H-120]^−^ and 215 [M−H-120−H_2_O]^−^ result from losses of C_4_H_8_O_4_ (120 *u*), C_3_H_6_O_3_ (90 *u*) and water molecules (18 *u*). The losses of 120 and 90 *u* are produced from the fragmentation of the sugar ring and suggest a *C*-hexoside group. All of these losses were confirmed by the calculation of the accurate masses of product ions. Thus, substances **10** and **11** were putatively identified as *C*-hexosyl- and *O*-pentosyl-5,7-dihydroxychromone, and their spectral data are similar to their reported data [30,31].

### 2.2. Chemical Composition 

The concentration of total phenols was 195.16 ± 0.94 mg GAE/g SAAE, and the concentration of flavonoids was 2.87 ± 0.08 mg QE/g SAAE. 

### 2.3. Antioxidant Activity 

#### 2.3.1. ABTS and DPPH Free-Radical Scavenging

The 50% inhibitory concentration (IC_50_) of 2,2′-Azino-bis(3-ethylbenzothiazoline-6-sulphonic acid) (ABTS) and 2,2-Diphenyl-1-picrylhydrazyl (DPPH) free-radicals as well as the maximum activity of the SAAE are outlined in Table 2. The SAAE had IC_50_ results similar to those of the antioxidant control, ascorbic acid, in both assays. Conversely, the necessary concentrations of SAAE to reach the maximum free-radical inhibition were two and five times higher than those of ascorbic acid in the ABTS and DPPH assays, respectively.

#### 2.3.2. Protective Effect of the SAAE Against Oxidative Hemolysis 

**Figure 3:**
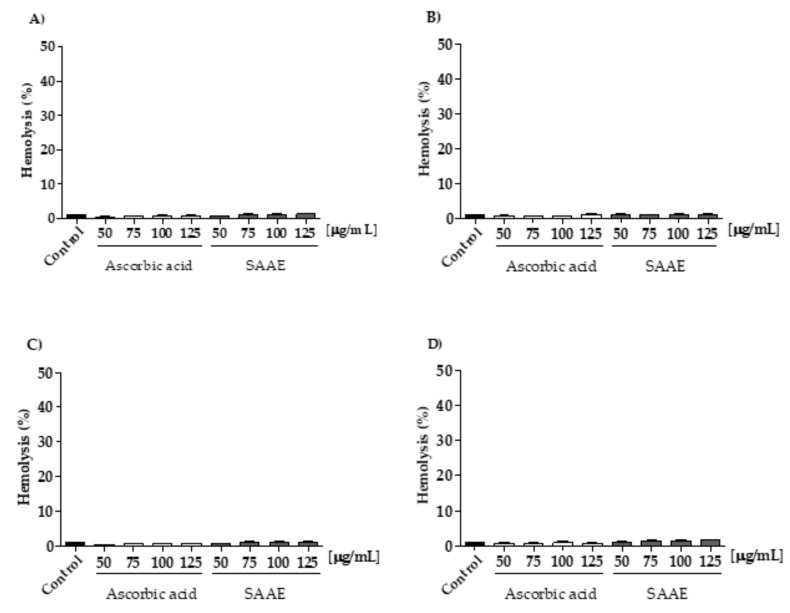
Assessment of hemolysis in human erythrocytes incubated for (**A**) 60, (**B**) 120, (**C**) 180 and (**D**) 240 min with different concentrations of ascorbic acid and the SAAE (50–125 μg/mL). Data are expressed as the mean ± SEM (*n* = 3) in duplicates.

**Figure 4:**
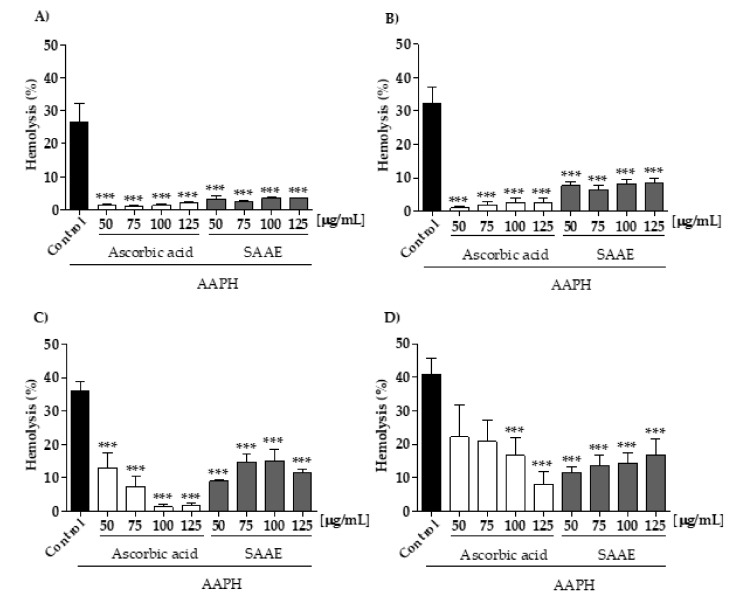
Assessment of hemolysis in human erythrocytes incubated for (**A**) 60, (**B**) 120, (**C**) 180 and (**D**) 240 min with the oxidizing agent AAPH with different concentrations of ascorbic acid and the SAAE (50–125 μg/mL). Data are expressed as the mean ± SEM (*n* = 3) in duplicates. *** *p* < 0.0001, compared with the control group AAPH.

Throughout the experimental period, the SAAE showed no hemolytic activity in human erythrocytes at any time and concentration tested (Figure 3A–D). When in contact with the oxidizing agent 2,2′-Azobis (2-methylpropionamidine) dihydrochloride (AAPH), all of the SAAE concentrations tested were able to protect the erythrocytes against hemolysis during the 240 min incubation in a concentration- and time-dependent manner (Figure 4A–D). This result was better than that of the antioxidant control, ascorbic acid, which lost its anti-hemolytic activity at concentrations of 50 and 75 μg/mL after 240 min of incubation (Figure 4D).

#### 2.3.3. Malondialdehyde (MDA) Dosage

**Figure 5:**
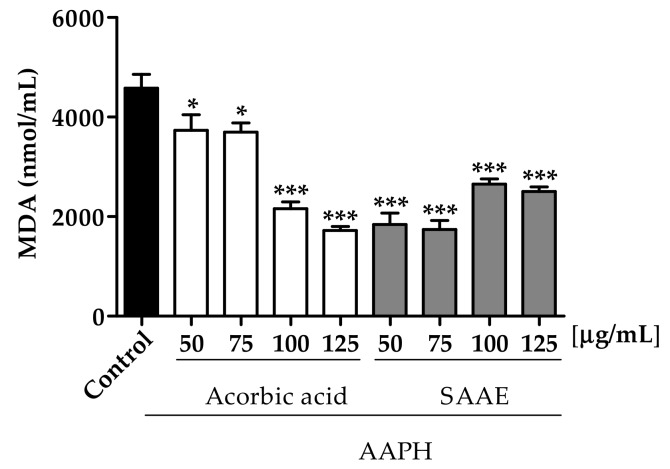
Malondialdehyde (MDA) concentration after adding the oxidizing agent AAPH to erythrocytes incubated for 240 min with different concentrations (50–125 μg/mL) of ascorbic acid and the SAAE. Data are expressed as the mean ± SEM (*n* = 3) in duplicate. * *p* < 0.05 and *** *p* < 0.0001 compared with the control group AAPH.

The ability of the SAAE to protect erythrocytes incubated with the oxidizing agent AAPH against lipid peroxidation was assessed by MDA quantification. All of the SAAE concentrations were able to reduce the MDA levels after 240 min of incubation. Erythrocyte treatment with the SAAE at concentrations of 50 and 75 μg/mL reduced the MDA levels by 59.9% and 62.0%, respectively, compared with the control AAPH. These results were better than those assessed with the control ascorbic acid, which showed similar reductions (52.8% and 62.3%) only at concentrations of 100 and 125 μg/mL, respectively (Figure 5). The higher doses SAAE were not so effective and a pro-oxidant effect probably occurs.

### 2.4. Cytotoxic Activity and Cell Death Profile

**Figure 6:**
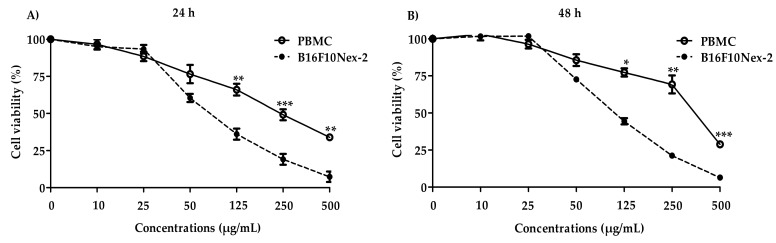
Viability curve in PBMC and B16F10Nex-2 cells. Cells were treated with different concentrations of the SAAE for (**A**) 24 h or (**B**) 48 h. Data are expressed as the mean ± SEM (*n* = 3) in triplicates. * *p* < 0.05, ** *p* < 0.01, and *** *p* < 0.0001 compared with B16F10Nex-2 cells.

B16F10Nex-2 cells were treated with the SAAE for 24 and 48 h to assess its cytotoxicity. Peripheral blood mononuclear cells (PBMC) were used as the control. Figure 6 shows that the extract had lower cytotoxicity against peripheral blood mononuclear cells (IC_50_ = 238.5 ± 32.1 µg/mL and IC_50_ = 257.7 ± 82.7 µg/mL) than against B16F10Nex-2 cells (IC_50_ = 65.0 ± 1.6 µg/mL and IC_50_ = 65.0 ± 5.6 µg/mL) in both treatment periods.

**Figure 7:**
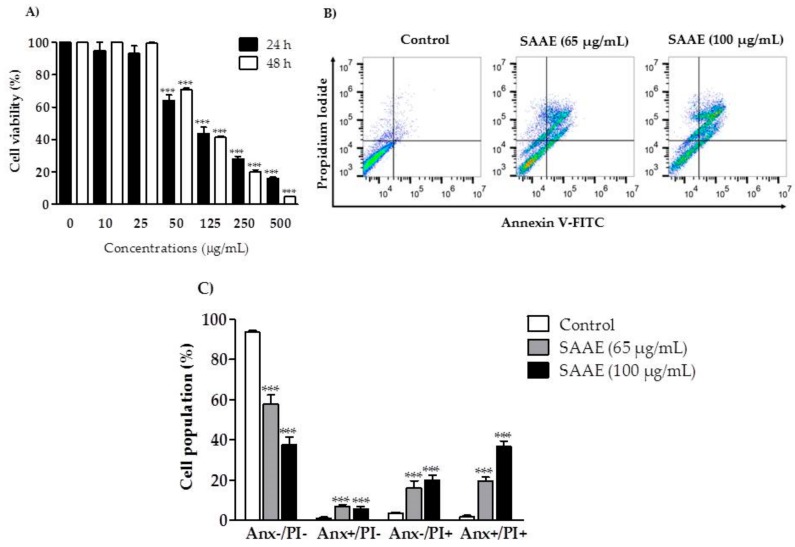
Viability and cell death profiles of the B16F10Nex-2 melanoma cell line after treatment with different concentrations of the SAAE. (**A**) Cell viability versus concentration (10–500 µg/mL) after 24 and 48 h of treatment. (**B**) Diagram of flow cytometry of cells stained with Annexin V–fluorescein isothiocyanate (FITC) and propidium iodide (PI) after a 24 h of treatment with the SAAE (65 and 100 µg/mL). The lower left quadrant (Anx^−^/PI^−^) represents viable cells, the lower right quadrant (Anx^+^/PI^−^) represents apoptotic cells, the upper left quadrant (Anx^−^/PI^+^) represents necrotic cells, and the upper right quadrant (Anx^+^/PI^+^) represents late apoptotic cells. (**C**) Percentage of dead cells assessed in the diagram corresponding to concentrations of 65 and 100 µg/mL. Data are expressed as the mean ± SEM (*n* = 3) in duplicates. *** *p* < 0.0001, compared with the untreated control group.

The SAAE showed concentration-dependent cytotoxic activity against B16F10Nex-2 cells (Figure 7A). After the 24 and 48 h treatments, the inhibitory concentration (IC_50_) observed was 65 µg/mL. Treatments with the SAAE at concentrations of 65 and 100 µg/mL promoted double labeling (annexin V^+^/PI^+^), highlighting cell death by late apoptosis in approximately 19.6 and 36.7% of cells, respectively (Figure 7B,C).

#### 2.4.1. Cell Cycle Phases

**Figure 8:**
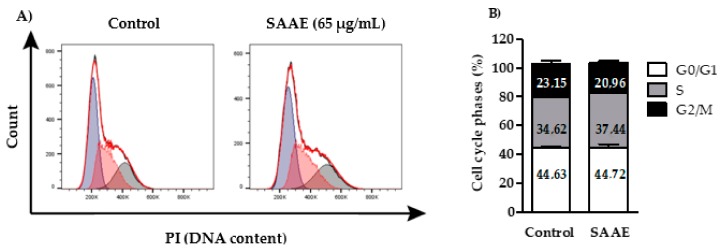
(**A**) Histogram and (**B**) graphical representation of the percentages of cells in the G0/G1, S and G2/M phases after treatment with the SAAE (65 μg/mL) for 24 h. Data are expressed as the mean ± SEM (*n* = 3) in duplicates.

The histogram shows the distribution of the cell cycle phases of control (untreated) and treated (65 µg/mL SAAE for 24 h) B16F10Nex-2 cells (Figure 8A). No significant differences were observed between cells from the control and SAAE treatment group, thus showing that treatment with the SAAE had no effect on the distribution of cell cycle phases of this cell line (Figure 8B)

#### 2.4.2. Reactive Oxygen Species (ROS) Levels

**Figure 9:**
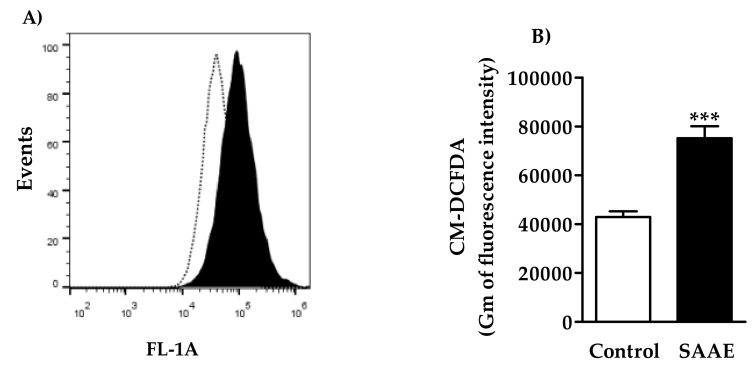
(**A**) Histogram and (**B**) graphical representation of the fluorescence intensity of ROS levels of B16F10Nex-2 cells treated with the SAAE (65 µg/mL). Data are expressed as the mean ± SEM (*n* = 3) in duplicates. *** *p* < 0.0001 compared with the untreated control group.

Cells treated with the SAAE (65 µg/mL) had increased ROS levels, as shown by the right-shifted fluorescence levels in the histogram (Figure 9A). The ROS levels increased by approximately 75% in B16F10Nex-2 cells treated with the SAAE in comparison to untreated cells (Figure 9B).

#### 2.4.3. Mitochondrial Membrane Potential

**Figure 10:**
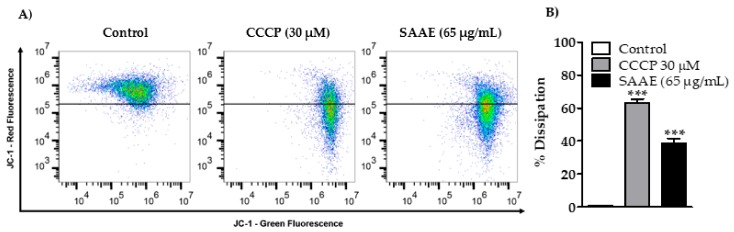
(**A**) Density Plot and (**B**) graphical representation of the percentages of mitochondrial membrane potential change in the control, CCCP-treated (30 μM) and SAAE-treated (65 μg/mL) B16F10Nex-2 melanoma cells. Data are expressed as the mean ± SEM (*n* = 3) in duplicates. *** *p* < 0.0001 compared with the untreated control group.

B16F10Nex-2 cells treated with the SAAE showed decreased mitochondrial membrane potential after 24 h of treatment (Figure 10A). The mitochondrial membrane potentials of cells treated with the positive control CCCP (30 μM) and the SAAE (65 μg/mL) decreased by 63.1 ± 2.4% and 38.7 ± 2.9%, respectively, in comparison with the untreated control cells (Figure 10B).

#### 2.4.4. Caspase-3 Activation

**Figure 11:**
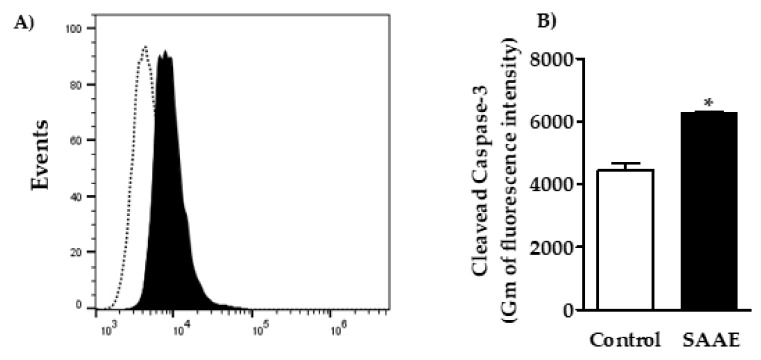
(**A**) Histogram and (**B**) graphical representation of caspase-3 activation in B16F10Nex-2 cells treated with the SAAE (65 μg/mL). Data are expressed as the mean ± SEM (*n* = 3) in duplicates. * *p* < 0.05 compared with the untreated control group.

The assessment of the effects of the SAAE on caspase-3 in B16F10Nex-2 cells showed a right shift in the fluorescence levels in the histogram (Figure 11A), with an approximately 40% increase in the cleaved caspase-3 labeling intensity compared with untreated control cells (Figure 11B).

## 3. Discussion

Medicinal plants have been used for centuries by humanity in the prevention and treatment of various diseases. However, the chemical constituents and biological properties of a wide variety of endemic plants in poorly studied biomes remain unidentified and uncharacterized. 

In this context, Brazil has been highlighted for its rich plant diversity with the potential for the development of new antioxidant and antitumor drugs, which are important for preventing oxidative stress-related diseases and different types of cancer [13]. 

*Stryphnodendron adstringens* is one of the species found in the Brazilian Cerrado that shows potential for the development of new drugs considering its chemical composition and biological activities previously described in the literature [3,5,6]. Previous phytochemical studies show that *S. adstringens* stem bark has proanthocyanidins [7,32], chalcones and triterpene compounds [4]. Those compounds are described for their antioxidant and anticancer activities [33,34,35,36]. 

In our study, gallic acid, gallocatechin, epigallocatechin, and dimeric as well as trimeric proanthocyanidins (condensed tannins), composed of mainly prodelphinidin along with procyanidin and prorobinetidin, were identified. These substances are commonly reported from *S. adstringens* [8,9]. However, chromone metabolites were only reported in the family Fabaceae [37]. Thus, the isomeric compounds *C*-hexosyl- and *O*-pentosyl-5,7-dihydroxychromone are newly discovered from the genus *Stryphnodendron*.

In recent years, natural antioxidants have gained importance for their prophylactic and therapeutic potential for many diseases as an effective tool in scavenging reactive species responsible for inducing oxidative stress [14,17,38]. The SAAE prepared from stem bark showed antioxidant activity by scavenging ABTS and DPPH free-radicals. This property is related to the concentration of phenolic compounds present in the SAAE, as these compounds are considered excellent natural antioxidants. These compounds are mainly described for their redox activity, acting as reducing agents, hydrogen donors and singlet oxygen scavengers [39], thereby preventing several diseases associated with oxidative stress [40]. Among the phenolic compounds identified in the SAAE, tannins are described as key antioxidant agents [41]. Furthermore, Luiz et al. [7] identified proanthocyanidins in *S. adstringens* stem bark, which are a class of polyphenols also known as condensed tannins. Dimeric procyanidins are the most active compounds of the proanthocyanidin class in scavenging free-radicals because they have a high molecular weight and a high degree of hydroxylation in the aromatic ring [41]. 

In addition to the direct effect on free-radical scavenging, the SAAE also protected human erythrocytes against damage caused by the antioxidant agent AAPH, resulting in oxidative hemolysis inhibition and reduced MDA levels. Antioxidant activity assessments in cellular models are highly important for studying the mechanisms of action of different compounds, including natural products. The antioxidant property observed in erythrocytes was not due to gene expression regulation because this cell model is anucleate, although it may occur through permeation of the compounds through the cellular membrane and interaction with endogenous antioxidant systems [42].

The ROS act as inducers of cell membrane lipid oxidation, a metabolic process that releases several by-products, such as malondialdehyde, which promotes DNA damage and may significantly contribute to cancer development [43]. 

The antioxidant capacity observed in erythrocytes may be related to the chemical compounds identified in the SAAE. Among the compounds identified, gallic acid is a well-described phenolic compound for its antioxidant and anti-hemolytic activities in human erythrocytes [14,44]. Procyanidins are also excellent antioxidants [45] capable of protecting erythrocytes from oxidative hemolysis and AAPH-induced lipid peroxidation [46,47]. Furthermore, flavonoids known as catechins are among the most abundant and important chemical constituents of green tea [48,49] and other plant species [13], and they are described as lipid peroxidation inhibitors [50]. 

Several studies have shown that extracts from natural products with high concentrations of phenolic compounds inhibit lipid peroxidation in human erythrocytes, thus decreasing the production of malondialdehyde [14,15] and showing cytotoxic effects on tumor cells [12,16,51]. 

The growing search for natural products with antioxidant properties and selective cytotoxic effects on tumor cells is among the strategies for discovering new anti-cancer drugs. Accordingly, this study aimed to investigate the cytotoxic activity of the SAAE against the B16F10Nex-2 melanoma line and to identify its mechanisms of action. The SAAE showed cytotoxic effects on melanoma cells, inducing cell death by apoptosis. Those effects may be attributed to the phenolic composition of the SAAE because these compounds have already been described for their cytotoxic effects on other tumor cell lines [52,53,54]. In vitro models are readily available and economically practical. In addition, they have been widely applied, including for studies related to discovery, therapeutic efficacy, and identification of molecular mechanism, optimizing the obtention of results. Despite the beneficial use of B16 lineage, melanoma cells isolated from human donors also are important to reveal the real clinical relevance of antitumor compounds [54].

Melanoma is among the cancers described for its chemoresistance to antitumor agents [55,56]. The search for derivatives of natural products has identified compounds with promising results in the treatment of this type of cancer. Among them, the chloroform fraction of *Anthelminticum centratherum* fruit seeds [57] and lectins extracted from *Polygonatum curtonema* rhizomes [58] showed cytotoxic effects via apoptosis in A375 melanoma cells resulting from increased ROS production, decreased mitochondrial membrane potential, cytochrome C release and caspase activation.

Apoptosis is a programed cell death process essential for maintaining homeostasis; however, this is the mechanism of action of some antitumor drugs, such as doxorubicin [59], cisplatin [60] and vinblastine [61]. This mechanism is characterized by a series of biochemical and morphological changes, including an increase in ROS levels [62], caspase activation [63], cell shrinkage, chromatin condensation, DNA fragmentation and the formation of apoptotic bodies [64]. ROS are chemically reactive molecules produced in the mitochondria that present essential functions in cellular respiration, acting in the maintenance of homeostasis in normal cells. In tumor cells, the ROS production imbalance may be irreversible, making the cell vulnerable to increased oxidative stress by promoting cellular apoptosis [65]. Agents such as procarbazine [66] and various phytochemical compounds have shown cytotoxic effects in tumor cells by increasing the intracellular ROS production [57,58,67]. The increase in the ROS promotes mitochondrial dysfunction, resulting in the loss of the mitochondrial membrane potential [68]. In this study, the SAAE increased the production of ROS, which may have triggered the dysfunction in mitochondrial membrane potential and apoptotic cell death observed in melanoma cells. Singh et al. [69] showed that proanthocyanidins extracted from cranberries have a cytotoxic effect on neuroblastoma cells by increasing ROS production and depolarizing the mitochondrial membrane potential. Furthermore, other extracts and natural compounds have already been described as promising in cancer treatment because they increase ROS and depolarize the mitochondrial membrane potential [70,71,72,73]. Excessive ROS production contributes to apoptotic cell death by releasing pro-apoptotic factors, including caspases [74]. The SAAE increased the caspase-3 activity, thus highlighting that the activation of this enzyme is directly involved in the apoptosis of B16F10Nex-2 melanoma cells.

Caspases are a family of proteases considered to be central regulators of apoptosis [75]. They act as a key apoptotic-signaling factor, triggered by the expression of proteins of the Bcl-2 family and by cytochrome C release from the internal mitochondrial membrane into the cytosol [76]. Caspases can be activated through two pathways, the extrinsic and intrinsic pathways [77]. The extrinsic pathway begins after extracellular signaling with death receptors [78], whereas the intrinsic pathway is initiated by intracellular stimuli that trigger mitochondrial membrane permeabilization, cytochrome C release into the cytosol, and subsequent caspase-9 activation, which activates caspase-3, the key enzyme for apoptosis [77,79,80].

Among the compounds identified, gallic acid shows cytotoxic effects against various tumor cell lines, including basal-like breast [81], non-small cell lung [82], oral squamous cell carcinoma [83] and hepatocellular cancer cell lines, showing no cytotoxic effects on normal cells [84]. In murine B16F10 melanoma cells, this compound is described for inducing apoptosis through the mitochondrial pathways by promoting the overexpression of the enzymes caspase-3, caspase-9 and PARP-1 as well as the pro-apoptotic proteins Bax and Bad; it also promotes the under-expression of the anti-apoptotic proteins Bcl-2 and Bcl-xL [85]. In human melanoma cells (A375.S2), in addition to the involvement of caspases and pro- and anti-apoptotic proteins, an increase in ROS production and a decrease in mitochondrial membrane potential are observed [86]. However, Badhani et al. [87] show that the anticancer potential of gallic acid is not related to its antioxidant activity but instead to its pro-oxidant activity. 

Prodelphinidins, whose subunits contain gallocatechin and epigallocatechin, inhibit cell proliferation and induce apoptosis in cancer cells by caspase-dependent extrinsic and intrinsic apoptotic pathways [88] as well as cell cycle arrest [89]. Procyanidins, the most abundant class of proanthocyanidins found in plants [88], show no cytotoxic effects on normal cells [90], although they are able to inhibit cell proliferation and induce apoptotic death in cancer cells by increasing the ROS levels [91], decreasing the mitochondrial membrane potential and activating caspase-3 [92]. Chromones and their derivatives are heterocyclic compounds distributed throughout the plant kingdom [93] known for presenting various pharmacological activities, including antioxidant and anticancer activities, especially against multiple drug-resistant tumor cell lines [94,95].

## 4. Materials and Methods 

### 4.1. Plant Material Collection

*Stryphnodendron adstringens* stem bark was collected at October 2014 and 2015 in the state of Mato Grosso do Sul, Brazil, at coordinates S 22°05′545, W 055°20′746, upon authorization from the SISBIO (Biodiversity Authorization and Information System), number 37931-3. 

The plant material was identified by a botanist of the School of Biological and Environmental Sciences-FCBA in Federal University of Grande Dourados-UFGD, Dourados, Mato Grosso do Sul (MS), Brazil. A voucher was deposited in the DDMS Herbarium under record number 4815.

### 4.2. Aqueous Extract Preparation 

Approximately 300 g of fresh bark was subjected to maceration in distilled water (2 L) for 48 h at room temperature. The macerate was filtered through Whatman No. 1 filter paper, frozen at −20 °C and subsequently lyophilized to prepare the *Stryphnodendron adstringens* aqueous extract (SAAE). The dry extract yield was 2.3%, calculated using the following formula: extraction yield (%) = (W_DE_/W_FB_) × 100, where W_DE_ is the dry extract weight (g) and W_FB_ is the fresh bark weight (g). The SAAE was stored at −20 °C and protected from light.

### 4.3. Chemical Analysis

#### 4.3.1. Identification of Constituents by LC-DAD-MS

A chromatographic system UFLC Shimadzu Prominence coupled to a diode array detector (DAD) and a mass spectrometer was used to analyze and identify the compounds from SAAE extract. The mass spectrometer was a MicrOTOF-Q III (Bruker Daltonics, Billerica, MA, USA), composed by an electrospray ionization source and quadrupole time-of-flight analyzers. The MS analyses were performed on negative and positive ion mode. The applied chromatographic and mass spectrometric parameters were the same described by Nocchi et al. [27]. The SAAE extract was solubilized in methanol and ultrapure water (6:4, *v*/*v*) at 1 mg/mL, filtered (PTFE membrane, 0.22 µm, Millex^®^, Millipore Corporation, Bedford, MA, USA) and 2 μL was injected on the chromatographic column.

#### 4.3.2. Determination of Phenolic Compounds and Total Flavonoids

##### Phenolic Compounds

To determine the total phenolic content present in the SAAE, the Folin–Ciocalteu colorimetric method was performed [96]. Thus, 2.5 mL of Folin–Ciocalteu reagent (1:10 *v*/*v*, diluted in distilled water) were added to 0.5 mL SAAE (at concentration 200 μg/mL). This solution was incubated in the dark for 5 min. Subsequently, 2.0 mL of 14% aqueous sodium carbonate (Na_2_CO_3_) were added and incubated at room temperature for 120 min and protected of light. The absorbance was measured at 760 nm using a T70 UV/Vis spectrophotometer (PG Instruments Limited, Leicestershire, UK). To construct a calibration curve, gallic acid (0.04–200 μg/mL) was used as standard. The concentration of phenolic compounds present in SAAE was expressed in mg equivalent to gallic acid (GAE)/g of SAAE. The assay was performed in triplicate.

##### Total Flavonoids

Total flavonoid content in SAAE was determined as described by Liberio et al. [97]. A methanolic solution of 2% aluminum chloride hexahydrate (AlCl_3_·6H_2_O) (4.5 mL) was added to 0.5 mL of SAAE (at concentration 200 μg/mL) and this solution was kept in the dark for 30 min at room temperature. Subsequently, the absorbances were measured at 415 nm (T70 UV/Vis spectrometer, PG Instruments Limited, Leicestershire, UK) and the flavonoid quercetin (00.4–200 μg/mL) was used to construct a calibration curve. The total flavonoid content was expressed in mg equivalent of quercetin (QE)/g extract. The assay was performed in triplicate. 

### 4.4. Antioxidant Activity

The antioxidant activities were determined by different methods and the doses were defined by previously assays performed, as well as based on data reported from species of Cerrado Biome [12,14,15,16].

#### 4.4.1. ABTS^•+^ Radical Discoloration Assay

The discoloration test of 2,2′-azinobis-(3-ethylbenzothiazoline-6-sulfonic acid) (ABTS^+^) radical was performed as described by Re et al. [98]. The ABTS^•+^ radical was prepared with 5 mL of ABTS^•+^ aqueous solution (7 mM) and 88 μL of potassium persulfate solution (140 mM). The solution was incubated for 12–16 h in dark at room temperature and then diluted in absolute ethanol (50 mL) to obtain an absorbance of 0.70 nm ± 0.05 units at 734 nm applying a T 70 UV/Vis spectrophotometer (PG Instruments Limited, Leicestershire, UK). A volume of 20 μL from SAAE solution (0.1–500 μg/mL) was mixed with 1980 μL of the ABTS^•^^+^ radical solution, incubated for 6 min (in the dark at room temperature) and the absorbances were measured at 734 nm. Ascorbic acid (AA) were used as a reference antioxidant and controls with the extract was performed for each concentration evaluated. Three independent assays were performed in triplicate. The percentage inhibition of the ABTS^•+^ radical was calculated according to the following equation, where Abs_control_ is the absorbance of ABTS^•+^ radical without the tested sample:

Inhibition of the radical ABTS^•+^ (%) = ((Abs_control_ − Abs_sample_)/Abs_control_) × 100

#### 4.4.2. DPPH Free Radical Capture Activity 

The 2,2-diphenyl-1-picrylhydrazyl (DPPH) radical capture activity was evaluated as described by Gupta and Gupta [99]. For the assay, 0.2 mL of SAAE extract (0.1–500 μg/mL) was added to 1,800 µL of DPPH solution (0.11 mM, 80% ethanol) and maintained for 30 minutes at room temperature in the dark. Thereafter, the absorbances at 517 nm were measured by a T 70 UV/VIS spectrophotometer (PG Instruments Limited, Leicestershire, UK). Ascorbic acid (AA) were used as a reference antioxidant and the controls of the extract were also acquired for each concentration evaluated. Three independent experiments were performed in triplicate. The inhibition was determined according to the following equation, where Abs_control_ is the absorbance of the unsampled DPPH solution:

Capture activity of DPPH (%) = (1 − Abs_sample_/Abs_control_) × 100

#### 4.4.3. Antioxidant Assay in Human Erythrocytes

##### Erythrocyte Suspension Preparation

The experiments were approved by the Research Ethics Committee (Comitê de Ética em Pesquisa, CEP) of the Federal University of Grande Dourados, (UFGD, Brazil (CEP process number: 2.407.793)). The human erythrocytes were obtained from peripheral blood of healthy donors. The collected blood was added in tubes containing the anticoagulant sodium citrate and they were centrifuged (400× *g* for 10 min). Subsequently, the plasma was removed, and the erythrocytes were washed three times with 0.9% sodium chloride solution (NaCl). The erythrocytes were resuspended in 0.9% NaCl solution, obtaining a final concentration of 2.5%.

##### Hemolytic Activity and Oxidative Hemolysis Inhibition

The evaluations of hemolytic activity and lipid peroxidation were determined in human erythrocytes according to the method described by Campos et al. [100]. The erythrocytes were preincubated with SAAE or ascorbic acid (50–125 μg/mL) at 37 °C for 30 min. Afterwards, 0.5 mL of 0.9% NaCl was added to evaluate the hemolytic activity of treatments or 0.5 mL of the oxidizing agent, 2,2′-azobis-(2-amidinopropane) dihydrochloride (AAPH), at 50 mM was added to evaluate the protection against oxidative hemolysis. As control of basal hemolysis, erythrocytes were incubated with 0.9% NaCl, while the erythrocytes were incubated with distilled water for total hemolysis control. The treatments were maintained for 240 min at 37 °C under constant agitation and analyzed each 60 min. To evaluate the hemolysis, the respective tubes (each time) were centrifuged at 700× *g* for 5 min and the supernatants (0.2 mL) were separated, added to 1800 µL of 0.9% NaCl and measured at 540 nm using a T 70 UV/VIS spectrophotometer (PG Instruments Limited, Leicestershire, UK). The percentage of hemolysis was calculated by the formula below; where *A* is the absorbance of sample and B the absorbance of the total hemolysis. Three independent experiments were performed in duplicate.

Hemolysis (%) = (A_sample_/B_total hemolysis_) × 100

##### Malondialdehyde (MDA) Dosage

The ability of SAAE to protect against lipid peroxidation in human erythrocytes was determined by dosage of malondialdehyde (MDA) according to the methodology described by Campos et al. [100]. Erythrocytes were preincubated with SAAE or ascorbic acid (50–125 μg/mL) at 37 °C for 30 min. Then, 0.5 mL of the oxidizing solution AAPH (50 mM) was added in each treatment and incubated at 37 °C for 240 min with constant stirring. After 60, 120, 180 and 240 min, the tubes were centrifuged at 700× *g* for 5 min, and then 0.5 mL of supernatant was transferred into tubes containing 1 mL of 10 nM thiobarbituric acid (TBA) and incubated at 96 °C for 45 min. Soon after, the samples were cooled, added n-butyl alcohol (4 mL) and centrifuged at 1600× *g* for 5min. The supernatant was measured at the wavelength 532 nm by a T 70 UV/VIS spectrophotometer (PG Instruments Limited, Leicestershire, UK). The control was prepared by the mixture of TBA (1 mL) and 20 mM MDA solution (0.5 mL). Two independent experiments were performed in duplicate. The MDA levels were expressed in nM/mL, according to the following formula:

$$\mathrm{MDA}\;(\mathrm{nmol}/\mathrm{mL})\;=\;\mathrm{Abssample}\;\times \;\left(\frac{20\;\times \;220,32}{\mathrm{AbsstandardMDA}}\right)$$

### 4.5. Cell Cultures

Human peripheral blood from healthy donors was collected in tubes containing the anticoagulant sodium citrate. Peripheral blood mononuclear cells (PBMC) was isolated by Ficoll Histopaque-1077 (1.077 g/cm^3^) (Gold Analisa Diagnóstica, Belo Horizonte, MG, Brasil) following the manufacturer’s instructions, the blood was centrifuged at 400× *g* for 30 min separating mononuclear cells into easily differentiated interfaces. The use of human samples was approved by the local Ethical Committee of the Federal University of Grande Dourados (protocol number 2.407.793) and signing written informed consent by donors.

The B16F10 cell line was originally provided by the Ludwig Institute for Cancer Research (LICR), São Paulo, Brazil. B16F10Nex-2, a sub-line isolated at the Department of Experimental Oncology, Federal University of São Paulo (UNIFESP), has the same characteristics as the original tumor cell line with moderate in vivo aggressiveness. Human peripheral blood mononuclear B16F10Nex-2 cells were grown in RPMI 1640 media (Gibco/Invitrogen, Minneapolis, MN, USA) supplemented with 10 nM 4-(2-hydroxyethyl) piperazine-1-ethanesulfonic acid (HEPES), 24 nM sodium bicarbonate and 10% fetal bovine serum (FBS), all from Gibco/Invitrogen, as well as with 40 mg/mL gentamicin (Hipolabor Farmacêutica, Sabará, Minas Gerais, MG, Brazil). All cells were kept in an incubator with a humidified atmosphere containing 5% CO_2_ at 37 °C.

#### 4.5.1. MTT Cell Viability Assay 

Cell viability was assessed based on the colorimetric assay using 3-(4,5-dimethylthiazol-2-yl)-2,5-diphenyltetrazolium bromide (MTT). PBMC (12 × 10^4^ cells/mL) and B16F10Nex-2 (5 × 10³ cells/mL) cells were plated in 96-well microtiter plates with different concentrations of the SAAE (0–500 µg/mL in RPMI 1640) for 24 and 48 h. After the treatments, the media were discarded, and 100 µL of MTT (0.5 mg/mL) was subsequently added to each well, followed by incubation for 4 h at 37 °C. After this period, the supernatant was discarded, and 100 µL of solvent (10% SDS, 0.01 M HCl and Milli-Q water) was subsequently added to solubilize the formazan crystals. The absorbance of each well was read at 630 nm using the SpectraMax 250 Microplate Reader (Molecular Devices, Sunnyvale, CA, USA). The cell viability was calculated using the following formula:

Cell viability (%) = (Abs_treated__cells_/Abs_control_) × 100

#### 4.5.2. Cell Death Profile

The cell death profile was evaluated according to the methods described by Paredes-Gamero et al. [101], with minor modifications. B16F10Nex-2 cells were grown on 48-well cell culture plates (1 × 10^4^ cells/mL) in RPMI medium containing 10% FBS for 24 h. After this period the cells were stimulated with IC50 of SAAE (65 μg/mL) and 100 μg/mL. Then, the cells were retired and washed with PBS. Afterward, the cells were resuspended in annexin labeling buffer (0.14 M NaCl, 0.01 M Hepes, and 2.5 Mm CaCl_2_, pH 7.4). The cells were labeled with annexin V-FITC and Propidium iodide (PI) (Becton Dickinson, San Jose, CA, USA) according to the manufacturer’s instructions for 15 min at room temperature. A total of 10,000 events were acquired per sample through analysis in an Accuri C6 flow cytometer (Becton Dickinson). The analysis was performed using FlowJo v10.2 LCC software (Ashland, OR, USA).

#### 4.5.3. Cell Cycle Phases

The cell cycle was profiled according to the method described by Paredes-Gamero et al. [101]. B16F10Nex-2 cells were plated in 24-well microtiter plates (6 x 10^4^ cells/mL) and grown in RPMI 1640 media supplemented with 10% FBS with or without SAAE (65 µg/mL) for 24 h at 37 °C. After this period, the cells were fixed, permeabilized as previously described and incubated with 4 mg/mL RNAse (Sigma-Aldrich) for 1 h at 37 °C. To label cellular DNA, the cells were incubated with 5 µg/mL SytoxGreen (Molecular Probes Inc., Eugene, OR, USA). The percentage of cells in each cell cycle phase (G0/G1, S and G2/M) was determined by flow cytometry, in an Accuri™ C6 cytometer (Becton Dickinson). A total of 20,000 events were acquired per sample. The analysis was performed using FlowJo v10.2 LCC software.

#### 4.5.4. Assessment of Reactive Oxygen Species (ROS) Levels

Intracellular ROS levels were assessed by flow cytometry using the dye 2′,7′-dichlorodihydrofluorescein diacetate (CM-H_2_DCFDA) (Molecular Probe-Life Technologies, Carlsbad, CA, USA). B16F10Nex-2 cells were plated in 24-well microtiter plates (6 × 10^4^ cells/mL) were treated with SAAE (65 µg/mL) for 24 h at 37 °C. Subsequently, the cells were trypsinized and incubated with 10 μM H_2_DCFDA for 30 min at room temperature and protected from light. The fluorescence of the ROS was assessed in an Accuri™ C6 flow cytometer (Becton Dickinson, San Jose, CA, USA). A total of 10,000 events were acquired per sample. The analysis was performed using FlowJo v10.2 LCC software.

#### 4.5.5. Assessment of the Mitochondrial Membrane Potential

To assess the possible effect of the SAAE on the mitochondrial membrane potential, B16F10Nex-2 cells were incubated with the fluorescent dye JC-1 (5,5’,6,6′-tetrachloro-1,1′,3,3′-tetraethylbenzimidazolylcarbocyanine iodide; Molecular Probes, Eugene, OR, USA) according to the method described by Moraes et al. [102]. The JC-1 probe accumulates in mitochondria depending on the potential. Viable cells have a high mitochondrial membrane potential and are labeled in red. When the mitochondrial membrane potential decreases, cells are labeled in green. In this assay, cells were plated in 24-well microtiter plates (6 × 10^4^ cells/mL) containing RPMI media supplemented with 10% FBS and treated with the positive control carbonyl cyanide m-chlorophenylhydrazone (CCCP, 30 μM) or with SAAE (65 µg/mL) for 24 h at 37 °C. After this period, the cells were trypsinized, centrifuged and incubated with JC-1 (1 µg/mL) for 15 min at room temperature. The fluorescence was analyzed in an Accuri™ C6 flow cytometer (Becton Dickinson). A total of 10,000 events were acquired per sample. The analysis was performed using FlowJo v10.2 LCC software.

#### 4.5.6. Caspase-3 Activity

Caspase-3 activation was assessed by flow cytometry according to the method described by Moraes et al. [102], with small modifications. B16F10Nex-2 cells were plated in 24-well plates (6 × 10^4^ cells/mL) containing RPMI media supplemented with 10% FBS and treated with SAAE (65 µg/mL) for 24 h at 37 °C. Then, cells were fixed with 2% paraformaldehyde in PBS for 30 min and permeabilized in 0.01% saponin for 20 min at room temperature. Subsequently, the cells were incubated with the antibody conjugated to the cleaved caspase-3 fluorophore (Asp175), Alexa Fluor 488, at room temperature and protected from light. After incubation, the fluorescence was analyzed in an Accuri™ C6 flow cytometer (Becton Dickinson, San Jose, CA, USA). A total of 10,000 events were acquired per sample. The analysis was performed using FlowJo v10.2 LCC software.

### 4.6. Statistical Analysis

Data are expressed as the mean ± standard error of the mean (SEM). Significant differences between groups were determined using the Student’s *t*-test for comparison between two groups and analysis of variance (ANOVA) followed by Dunnett’s test for comparison of two or more groups using the GraphPad Prism 5 software (San Diego, CA, USA). The results were considered significant when *p* < 0.05.

## 5. Conclusions

In conclusion, the results from the present study show that the SAAE from stem bark had a high concentration of phenolic compounds (flavan-3-ols, gallic acid, proanthocyanidins and chromones), which may be related to its antioxidant activities and anticancer effects on B16F10Nex-2 melanoma cells. Furthermore, the SAAE promoted cell death by apoptosis by increasing the intracellular ROS levels, mitochondrial membrane potential dysfunction and cleaved caspase-3 activation. These results open new perspectives for studies on these effects on other tumor cell lines and in vivo cancer models.

## Figures and Tables

**Table 1:** Identification of the constituents from *S. adstringens* aqueous extract (SAAE) by LC-DAD-MS/MS.

Peak	RT (min)	Compound	UV (nm)	MF	Negative Mode (*m/z*)		Positive Mode (*m/z*)	
					MS [M − H]^−^ (*)	MS/MS	MS [M + H]^+^ (*)	MS/MS
1	1.2	PDE-PDE (B type)	275	C_30_H_26_O_14_	609.1250 (2.5)	423, 305, 177	611.1411 (2.6)	287, 263, 179
		PDE-PDE-PDE (B type)		C_45_H_38_O_21_	913.1803 (3.3)	423, 305, 261, 243, 177	915.1955 (2.5)	-
		di-hexoside		C_12_H_22_O_11_	341.1093 (1.0)	-	365.1051 (0.9)	-
2	2.4	Gallic acid ^st^	270	C_7_H_6_O_5_	169.0140 (1.7)	-	171.0291 (1.9)	-
3	2.9	PDE-PDE (B type)	275	C_30_H_26_O_14_	609.1280 (4.9)	423, 305, 177, 165	611.1392 (0.6)	425, 299, 287, 275, 263, 179
4	3.6	PDE-PDE (B type)	276	C_30_H_26_O_14_	609.1254 (0.8)	423, 305, 177, 165	611.1395 (1.6)	425, 299, 287, 263, 179
5	4.2	PDE-PDE (B type)	275	C_30_H_26_O_14_	609.1259 (1.6)	423, 305, 177, 165	611.1400 (0.7)	425, 299, 287, 275, 263, 245, 179
6	4.3	Gallocatechin ^st^	275	C_15_H_14_O_7_	305.0673 (2.0)	179	307.0812 (3.2)	163, 159
7	6.1	PDE-PDE (B type)	275	C_30_H_26_O_14_	609.1245 (0.8)	-	611.1422 (4.3)	-
8	10.3	Epigallocatechin ^st^	274	C_15_H_14_O_7_	305.0673 (1.9)	167	307.0823 (3.4)	195, 177, 163, 159
9	13.5	PRO-PDE (B type)	280	C_30_H_26_O_13_	593.1312 (1.9)	305, 177	595.1446 (0.1)	427
10	15.5	*C*-hexosyl *O*-pentosyl 5,7-dihydroxychromone	257, 285, 327 ^sh^	C_21_H_26_O_13_	485.1312 (2.4)	353, 335, 245, 233, 215, 205	487.1460 (2.9)	355, 337, 319, 289, 259, 235, 205
11	17.3	*C*-hexosyl *O*-pentosyl 5,7-dihydroxychromone	257, 295, 325 ^sh^	C_21_H_26_O_13_	485.1317 (3.3)	365, 353, 335, 263, 245, 263, 233, 215, 205	487.1452 (1.0)	319, 301, 283, 259, 235, 205
12	19.0	PCY-PDE (B type)	278	C_30_H_26_O_13_	593.1308 (1.3)	305	595.1453 (1.1)	427, 307, 289
13	19.8	PDE-PDE benzoate (B type)	278	C_37_H_30_O_16_	729.1461 (3.5)	423, 305, 287, 261, 177	731.1611 (0.6)	425, 407, 299, 287, 275, 263
14	23.5	NI	280	C_26_H_32_O_12_	535.1840 (3.6)	-	537.1981 (2.7)	-
15	35.4	NI	290, 330	C_32_H_36_O_16_	675.1946 (2.2)	245, 233, 215, 207	677.2088 (1.8)	235, 191, 163
16	36.2	NI	300	C_33_H_38_O_17_	705.2068 (4.5)	573, 467, 365, 335, 317, 245, 237, 233, 215	707.2193 (1.6)	325, 221, 191

*: error in ppm; ^st^: confirmed by authentic standard; ^sh^: shoulder; MF: molecular formula; RT: retention time; NI: non identified; PDE: prodelphinidin, PRO: prorobinetidin; PCY: procyanidin.

**Table 2:** IC_50_ and maximum activity of the standard antioxidant and of *S. adstringens* aqueous extract (SAAE) in ABTS and DPPH free-radical scavenging.

Methods	Ascorbic Acid			SAAE		
	IC_50_	Maximal	Inhibition	IC_50_	Maximal	Inhibition
	µg/mL	%	µg/mL	µg/mL	%	µg/mL
ABTS	1.34 ± 0.01	99.67 ± 0.04	5	1.83 ± 0.15	99.68 ± 0.08	10
DPPH	2.65 ± 0.03	87.44 ± 2.13	10	3.81 ± 0.02	89.92 ± 1.36	50

Results are expressed as the mean ± SEM (*n* = 3).

## Abbreviations

AAPH	2,2’-Azobis (2-methylpropionamidine) dihydrochloride
ABTS	2,2’-Azino-bis(3-ethylbenzothiazoline-6-sulphonic acid)
CCCP	Carbonilcianeto-m-clorofenilhidrazona
CM-H2DCFDA	2′,7′-dichlorodihydrofluorescein diacetate
DPPH	2,2-Diphenyl-1-picrylhydrazyl
FBS	Fetal bovine serum
GAE	Gallic acid equivalente
HEPES	4-(2-Hydroxyethyl) piperazine-1-ethanesulfonic acid
LICR	Ludwig Institute for Cancer Research
MDA	Malondialdehyde
MTT	3-(4,5-dimethylthiazol-2-yl)-2,5-diphenyltetrazolium bromide
NaCl	Sodium chloride
PBMC	Human peripheral blood mononuclear cells
PI	Propidium iodide
QE	Quercetin equivalents
ROS	Reactive oxygen species
SAAE	*Stryphnodendron adstringens* aqueous extract
SEM	Standard error of the mean
TBA	Thiobarbituric acid
